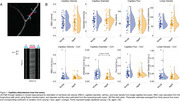# The aging vasculature in hippocampus

**DOI:** 10.1002/alz70855_105519

**Published:** 2025-12-24

**Authors:** Signe H. Mikkelsen, Oliver Bungaard Christensen, Dmitry Postnov, Leif Østergaard, Eugenio Gutiérrez‐Jiménez

**Affiliations:** ^1^ CFIN, Department of Clinical Medicine, Aarhus University, Aarhus, Denmark; ^2^ Aarhus University Hospital, Aarhus, Denmark; ^3^ Department of Biomedicine, Aarhus University, Aarhus, Denmark

## Abstract

**Background:**

The vascular network in the brain delivers oxygen and nutrients to cells to maintain normal brain function. Substrate delivery depends on local cerebral blood flow (CBF) regulation and capillary transit‐time distribution. Damage to capillaries and cells regulating capillary flow dynamics may impair blood flow control and increase capillary transit‐time heterogeneity (CTH), limiting oxygen availability to areas of high metabolic demand. These changes are particularly important in the hippocampus, which has distinct vascular topography compared to cortex and plays a key role in cognition. Age‐related vascular dysfunction often precedes cognitive impairment yet remains poorly understood in the hippocampus. Further studies are necessary to explore how vascular changes contribute to the development of Alzheimer's disease, where the hippocampus is one of the first areas affected. This study aims to investigate hippocampal capillary flow dynamics and oxygenation in aging.

**Method:**

Imaging was performed with awake‐restrained in vivo two‐photon microscopy (TPM) and laser speckle contrast imaging (LSCI) through a chronic hippocampal cranial window in young and aged female mice. Steady state hippocampal hemodynamics were investigated using intravascularly (IV)‐administered fluorescent dyes. CTH was estimated using an indicator dilution technique. IV partial pressure of oxygen (pO2) and tissue oxygen tension (PtO2) estimates were achieved using oxygen‐sensitive dye injected IV and through a ventricular cannula, respectively. Pulsatility index (PI) was calculated from LSCI scatter patterns without contrast agents. A spatial learning and memory assay evaluated cognitive impairment.

**Result:**

We will present an analysis of age‐related changes in hippocampal microvascular hemodynamics. Preliminary data suggest capillary diameter estimates in aging mice appear less dynamic than in youth (Figure 1), while flow dynamics remain unchanged.

**Conclusion:**

Further analysis is expected to reveal changes in pO2 corresponding to altered capillary flow distributions to build on preliminary observations. Correlating vascular and behavioral changes will provide a broader understanding of age‐induced cognitive changes.